# An overview of healthcare-associated Candida auris outbreaks in Ministry of Health hospitals–Saudi Arabia 2020–2022; Retrospective multicentric study

**DOI:** 10.1371/journal.pone.0313589

**Published:** 2025-01-03

**Authors:** Khalid H. Alanazi, Hala M. Roushdy, Waad I. ALzubaidi, Arwa S. Alanazi, Nawal M. Alanazi, Ahlam H. Alamri, Yahya I. Alnshbah, Nawaf M. AL Almatrafi, Zainah M. Al Shahrani, Adel S. Alanazi, Nasser H. Alshanbari

**Affiliations:** 1 General Directorate of Infection Prevention & Control, Ministry of Health-Saudi Arabia, Riyadh, Saudi Arabia; 2 Research and Scientific Center in Prince Sultan bin Abdulaziz Humanitarian City, Riyadh, Saudi Arabia; Universidad Autonoma de Chihuahua, MEXICO

## Abstract

**Background:**

*Candida auris* (C. auris) is an emerging fungus pathogen associated with nosocomial infections that is seen as a serious global health issue.

**Aim:**

To describe the epidemiology and features of hospital-acquired *Candida auris* outbreaks in the Ministry of Health hospitals (MOH).

**Methods:**

A three-year (2020–2022) retrospective analysis of *Candida auris* outbreaks in the Saudi MOH hospitals. A total of 45 hospitals were involved, with 511 cases of *Candida auris*. The data collected from the General Directorate of Infection Prevention and Control (GDIPC) platform comprises all patients’ data for *C*. *auris* instances, whether infected or colonized.

**Results:**

Out of the 511 Candida auris recruited cases, 291 (56.9%) were infected, whereas 220 (43.1%) were colonized. 32.9% of cases were above the age of 65, and 68.9% were male. The majority of cases were admitted in the ICU unit (95.5%). Approximately 18.8% of cases were diabetes, and 18.5% were hypertensive. Central lines were discovered in 37.7% of the cases. Approximately 85.9% of cases had Foley catheters, 68.5% were on ventilators, and 53.8% had a tracheostomy. The crude mortality rate was particularly high (41.5%) among the study cases. It was non-significantly higher among infected individuals (44.7%) than colonized patients (37.3%) at (*p value* = 0.093). The prevalence of hypertension (p = 0.001), DM (p = 0.003), and peripheral line insertion (p = 0.004) was significantly higher among colonized patients. While the presence of COVID-19 (p = 0.001), and central line insertion (p = 0.001) was significantly higher among infected patients.

**Conclusion:**

*C*. *auris* is a new pathogen that causes hospital outbreaks. Strict adherence to infection prevention and control criteria established by the Centre for Disease Control (CDC) and GDPIC has significantly contained and reduced the spread of these outbreaks. One-month retrospective surveillance before diagnosing the index case and a prospective surveillance strategy for at least three months is highly recommended.

## Background

*Candida auris* is a multidrug-resistant yeast that produces fatal invasive infections [1, 2]. *C*. *auris*, a unique Candida species isolated from the external ear canal of a patient in Japan, was described for the first time in 2009 [3].

The most recent World Health Organization list of fungal priority diseases includes *C*. *auris* as a critical pathogen [4]. Infections with *C*. *auris* have been recorded often from the circulation, as well as in conjunction with CSF [5]. It’s also been found in wounds, ear, and respiratory samples, as well as urine, and bile. Detection in axillary and groin monitoring swabs may suggest carrier rather than infection, with carriage considered a risk of transmission to others and potential invasive infection [6].

Compared to other Candida species, *Candida auris* identification necessitates specialized laboratory procedures. This could lead to problems with identification, epidemic detection, and control. *C*. *auris* appears highly transmissible between patients in healthcare settings via contaminated environments or equipment, comparable to but distinct from other Candida species. It is also associated with long-term environmental persistence [6, 7]. *C*. *auris* can thrive at greater temperatures and survive high salt concentrations than other fungi [6, 8, 9]. These are crucial qualities in its ability to survive in the environment for lengthy periods [10–12].

Severe underlying disease with immunosuppression, bone marrow transplantation, corticosteroid therapy, neutropenia, malignancy, chronic kidney disease or diabetes mellitus, a prolonged stay in ICU, mechanical ventilation, presence of a central venous catheter or urinary catheter, prolonged exposure to broad-spectrum antibiotic or antifungal use, underlying respiratory illness, vascular disease are the most common risk factors for *C*. *auris* acquisition in health care settings [6, 12].

Overall mortality from *C*. *auris* infection is reported to be high in the literature, ranging from 40–60% worldwide, possibly due to severe underlying conditions in at-risk populations, the pathogen’s multi-drug resistance, and the limited availability of certain antifungal drugs in some countries [13, 14].

Rapid and accurate identification of hospitalized patients infected/colonized with *C*. *auris*, rapid detection of susceptibility patterns, and appropriate use of infection control measures can help to contain the spread of this highly pathogenic yeast in healthcare settings and prevent/control outbreaks. Early detection of *C*. *auris* infections is advantageous, with earlier beginning of proper antifungal therapy saving many lives [15].

Up to the authors’ knowledge, only a few research studies have been published to describe *C*. *auris* outbreaks in Saudi Arabia, and they have all been limited to a single epidemic in a single healthcare environment. There have also been no published study articles covering all MOH hospitals in Saudi Arabia, neither to characterize the present state and numbers of confirmed *C*. *auris* outbreaks nor to identify the common infection control risk factors associated with the development of such outbreaks. So, we intended to manage these research gaps for further in-depth improvement in utilizing epidemiological laboratory and clinical surveillance data, as well as other health care data, to quantify the burden of Invasive Fungal Disease (IFD) and antifungal resistance to inform public health interventions, guide IPC measures, and ultimately improve the quality of the Healthcare provided to Patients in the Saudi MOH Hospitals. The current investigation aimed to describe the characteristics of HA *Candida auris* outbreaks in Saudi Hospitals and to evaluate the infection control measures and their role in the containment of these outbreaks.

## Patients and methods

### Study design, duration, and settings

A three-year retrospective analysis of *Candida auris* outbreaks and cases in Saudi MOH hospitals (2020–2022). During the analysis, 511 cases of *Candida auris* were discovered in 45 MOH hospitals. All the included hospitals were tertiary hospitals with a bed capacity ranging from 100 to 300 beds.

### Case definitions

According to CDC candida auris 2019 case definition [16].

### Candida auris colonization

**Probable**: Person with presumptive laboratory evidence from a swab collected for the purpose of screening for C. auris colonization regardless of site swabbed.

**Confirmed**: Person with confirmatory laboratory evidence from a swab collected for the purpose of screening for C. auris colonization regardless of site swabbed. Typical colonization/screening specimen sites are skin (e.g., axilla, groin), nares, rectum, or other external body sites.

### Candida auris infection

**Suspected case:** Person with presumptive laboratory evidence from a clinical specimen collected for the purpose of diagnosing or treating disease in the normal course of care and no evidence of epidemiologic linkage. A clinical specimen includes specimens from sites reflecting invasive infection (e.g., blood, cerebrospinal fluid) and specimens from non-invasive sites such as wounds, urine, and the respiratory tract, where presence of C. auris may simply represent colonization and not true infection.

**Probable case:** Person with presumptive laboratory evidence from a clinical specimen collected for the purpose of diagnosing or treating disease in the normal course of care and evidence of epidemiologic linkage.

**Confirmed case**: Person with confirmatory laboratory evidence from a clinical specimen collected for the purpose of diagnosing or treating disease in the normal course of care.

#### Candida auris outbreak definition

A Candida auris HAI outbreak is typically defined by the Saudi MOH as the occurrence of two or more cases of C. auris infections or colonization that are epidemiologically linked to the location, exposure, and duration [17].

### Candida auris identification system

✓ **Specimen selection and collection**
• According to the policy of the National Center for Disease Prevention and Control [18], suspected case identification begins in the microbiology laboratory for any patient who has a positive culture for *Candida auris* from any form of body sample (blood, sputum, wound swab, etc.).
• All specimens must be accompanied by the referral form filled with the patient’s name, sex, age, specimen source, and patient history.
✓ **Specimen processing**
• C. auris grows on Blood agar as all other Candida. species. But for sub-culturing, we use Sabouraud’s agar. Growth at 40-42oC is useful to differentiate it from many other Candida Species. CHROM agar is widely used as a differentiation medium; C. auris appears in pale purple or pink colonies.
• Microscopically it is indistinguishable from other Candida species, but it is a germ tube-negative budding yeast.
• Until recently, automated yeast identification systems like Vitek2 (Vitek2 YST) routinely misidentified C. auris isolates as C. haemulonii or Rhodotorula glutinis. However, Vitek2 YST with upgraded software (version 8.01, which includes C. auris and other automated yeast identification systems) now usually correctly identifies C. auris. The former was used to test the majority of the specimens in MOH hospital laboratories.
• In laboratories without updated identification databases in their diagnostic devices, referral of Candida non-albicans and invasive isolates to a reference laboratory was done. A confirmatory test by VITEK MS (bioMerieux, France) and MALDI-TOF Biotype system (Bruker Corporation, USA) or by DNA sequencing present in reference labs.
✓ **Antifungal susceptibility testing for Candida auris positive specimens**

The Clinical and laboratory standards institute (CLSI) recommended broth microdilution (BMD) method for azoles (fluconazole, and voriconazole), amphotericin B, and echinocandins (caspofungin and micafungin) was applied to all isolates that were confirmed to be C. auris. Breakpoints were established according to expert opinion as published by the US Centers for Disease Control and Prevention (CDC) [19]. The threshold for resistance to azoles was determined at ≥32 μg/ml. Additionally, resistance to ≥2 μg/ml amphotericin B was established. Micafungin resistance was set at ≥4 μg/ml and capsofungin resistance at ≥2 μg/ml.

✓ **Reporting Confirmed cases of C. auris**

According to our policy, any confirmed cases of C. auris were reported with its susceptibility testing to the General Directorate of Infection Prevention and Control (GDIPC) through the national approved electronic platform. All reports should be generated within 24 hours of identification through the GDIPC healthcare-associated infections (HAIs) outbreak notification electronic platform.

### Data collection and analyses

• Data gathered from the GDIPC platform involving all demographic patient data for *C*. *auris* cases, whether infected or colonized, as well as underlying conditions, risk factors, and death. Data access was started on December 1, 2023. Incomplete records with missing data were excluded from the analysis.

• The subjects were coded anonymously with serial numbers for data analysis. All statistical analyses were performed using the SPSS software "IBM SPSS Statistics for Windows, version 25 (IBM Corp., Armonk, N.Y., USA)". All qualitative variables were subjected to frequency analysis. For quantitative data, mean and standard deviation were calculated. For univariate analysis, the Chi-square test was employed to identify indicators that had a significant relationship with the patient’s risk variables.

### Ethical considerations

The study was carried out following Helsinki principles. The GDRS (General Directorate of Research and Studies) of the Ministry of Health had granted IRB permission. Because of the nature of the research, informed consent has been waived. Except for the principal investigators, the identities, medical record numbers, and hospital names of the participants were kept confidential and inaccessible.

## Results

**Fig 1:**
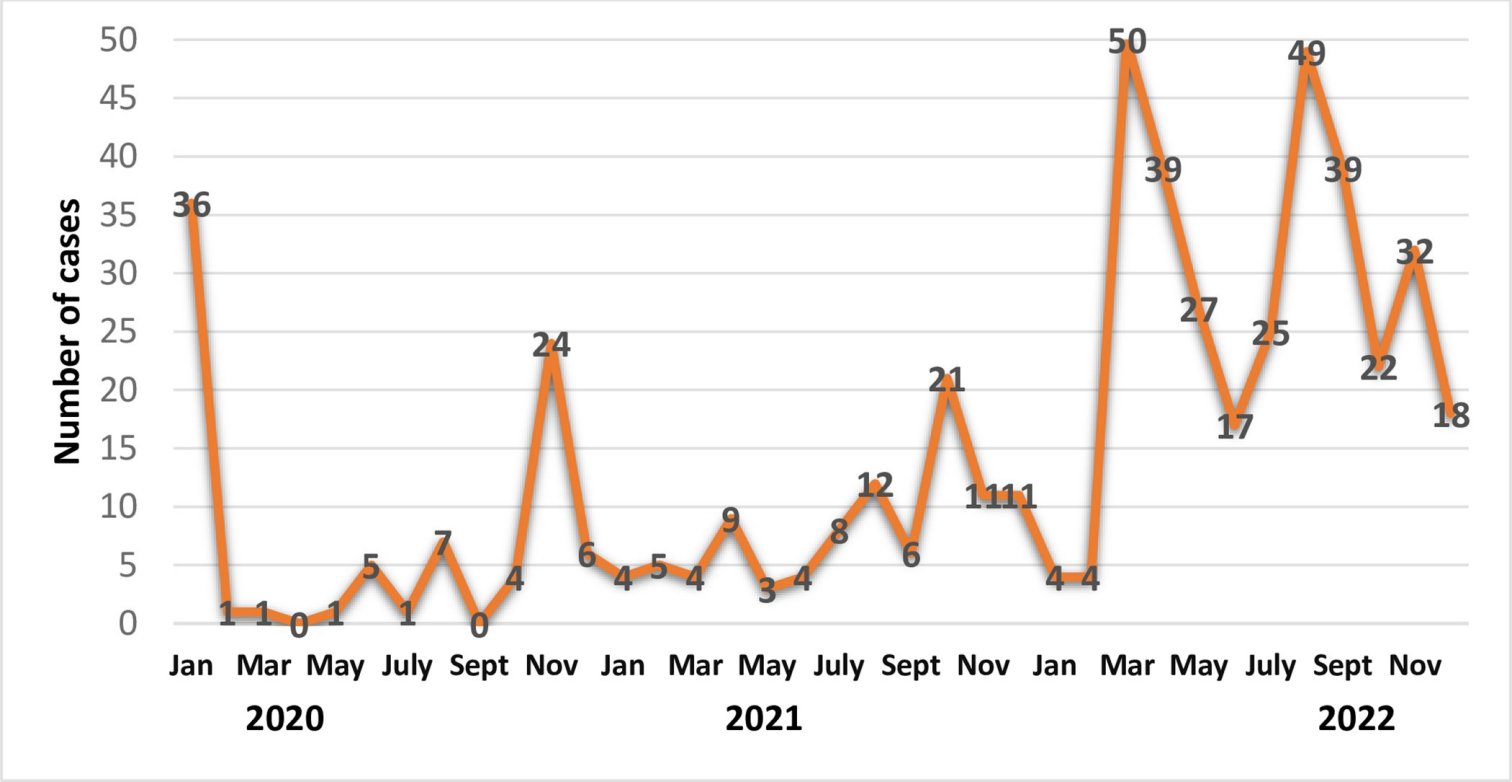
Number of *Candida auris* cases according to the date of the report.

186 *Candida auris* outbreaks were documented in Saudi Arabia’s Ministry of Health facilities in the current study including 511 patients. In 2020, there were 22 outbreaks with 84 cases of *Candida auris*. There were 28 outbreaks with 96 cases in 2021. And 136 outbreaks with 331 cases in 2022. Fig 1 depicts the overall number of cases per month for the three years of the study. The number of cases reached 36 in January 2020, then dropped to less than 10 per month in the following months. It grew a second time in November 2020, reaching 24 cases. The total number of cases in 2021 ranged from 4 to 21. The year 2022 saw the greatest number of cases ranging from 17 to 50.

**Fig 2:**
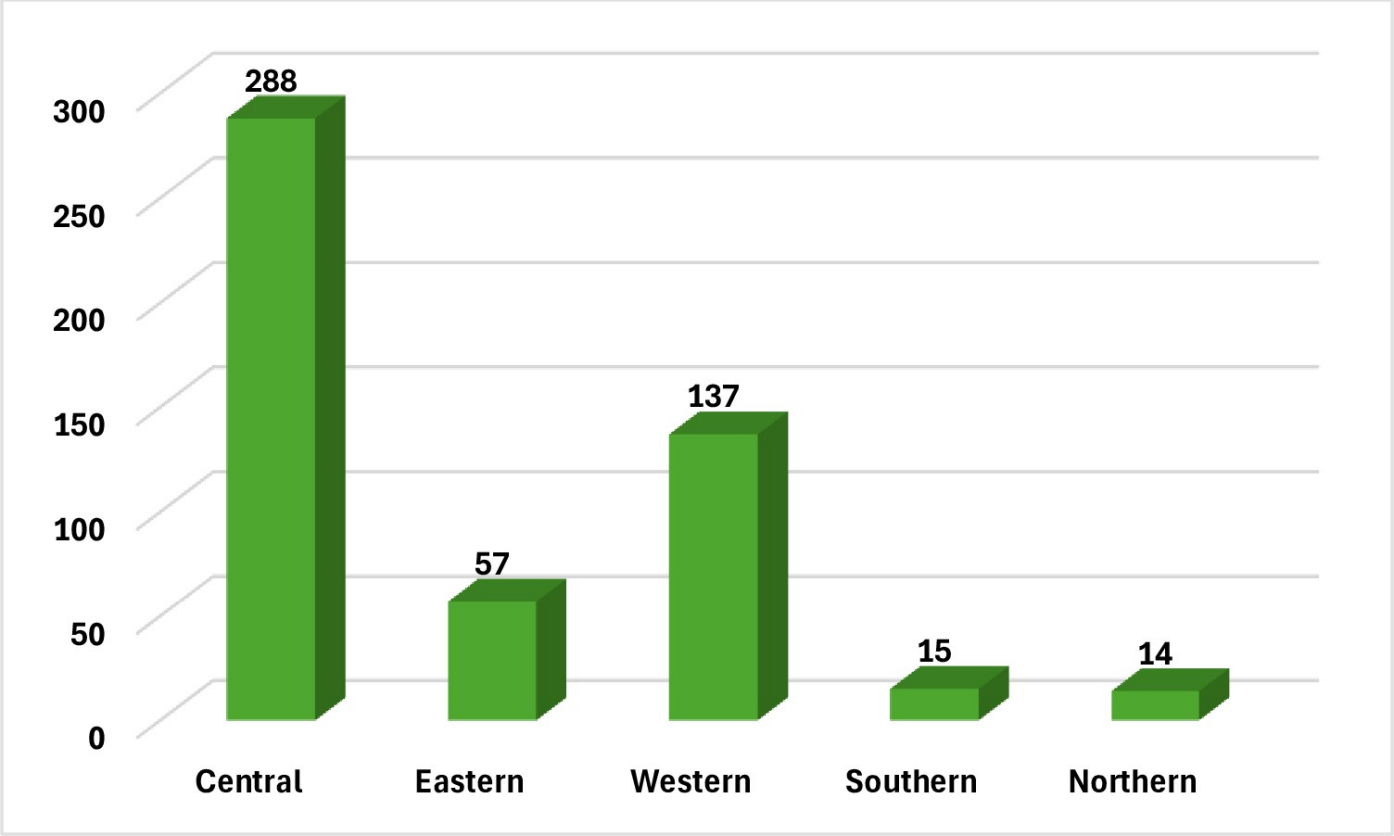
Distribution of Candida auris cases according to the provinces in the Kingdom.

Fig 2 shows that the central province had the highest prevalence of cases (n = 288), spread over 7 hospitals, followed by the western province (n = 137) throughout 14 hospitals. In the eastern province, 137 cases were present among nine hospitals. In the southern province, 15 cases were distributed in two hospitals. And in the northern province, 14 cases are distributed in six hospitals.

**Fig 3:**
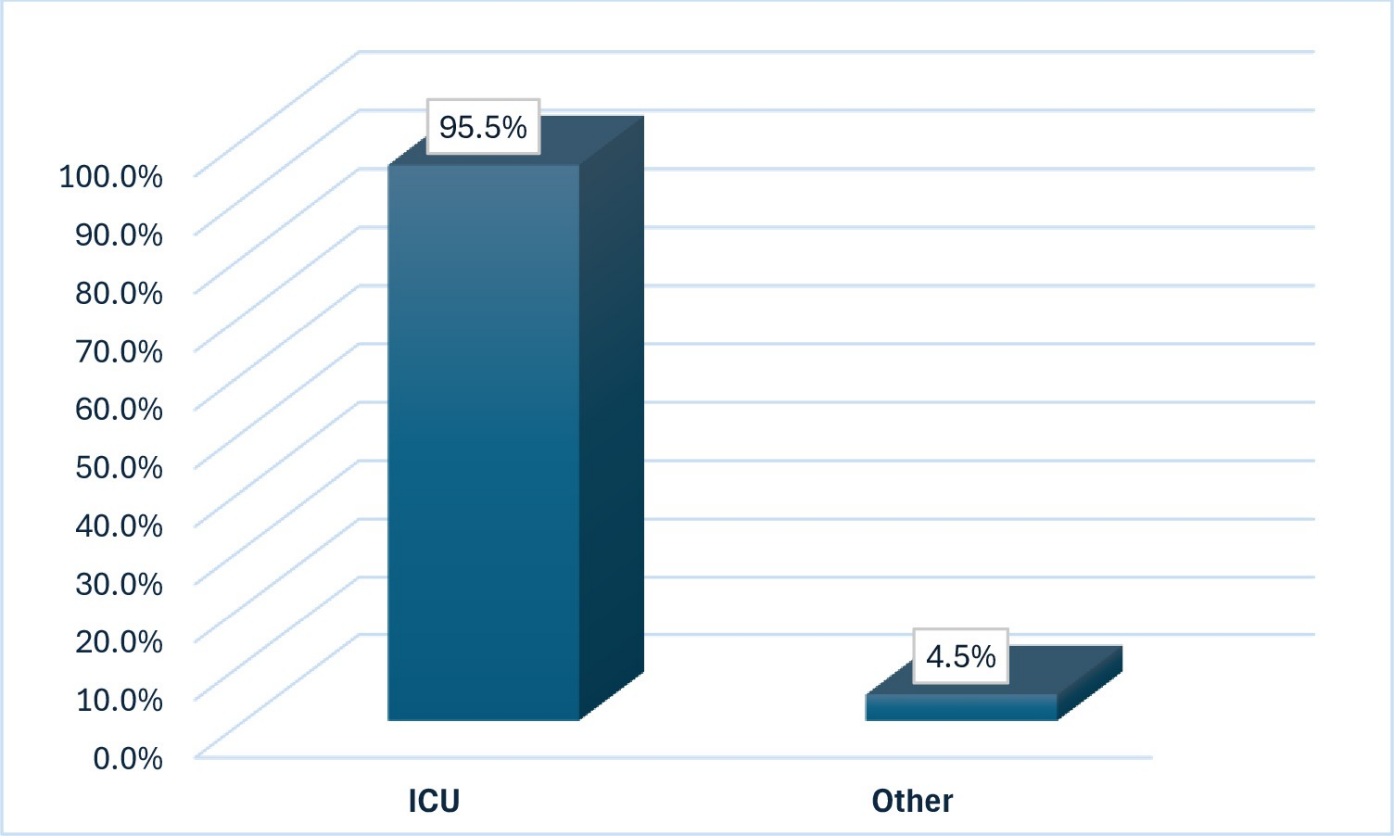
Distribution of the studied cases regarding their hospital admission unit.

During the three years of the study, 511 patients were enrolled. The majority were above 65 years old (32.9%) and males (68.9%). About 46.2% of cases had no risk factor, 18.8% were diabetics, and 18.5% were hypertensive. Out of 511 cases, 291 of the cases were infected with *Candida auris* species (56.9%) and 220 cases were colonized (43.1%). Colonization sites are axilla, groin, and nasal in 166 cases, urine in 43 cases, wound in 6 cases, and sputum in 5 cases. Swabs (36.4%) were the most commonly utilized sample type for diagnosis, followed by blood (35.6%). Central Line was found in 37.7% of the instances. Approximately 85.9% of cases had Foley catheters, 68.5% had ventilators, and 53.8% had tracheostomy. Moreover, two-thirds of them (85.3%) had used antibiotics. The central region had the highest percentage of analyzed cases (56.4%), followed by the western region (26.8%). The majority of cases were admitted in the ICU (n = 488, 95.5%), **Table 1 and Fig 3.**

**Table 1:** Baseline characteristics of the studied *Candida auris* cases.

		Total N = 511 (%)	Year		
			2020 N = 76	2021 N = 95	2022 N = 328
**Age in years** *	Mean (±SD)	51.5 (±23.3)	46.6 (±24.5)	51.9 (±23.4)	52.6 (±22.8)
	≤45	191 (38.2)	26 (13.6)	40 (20.9)	125 (65.5)
	46–64	144 (28.9)	28 (19.4)	22 (15.3)	94 (65.3)
	≥65	**164 (32.9)**	22 (13.4)	33 (20.1)	109 (66.5)
**Gender**	Male	**352 (68.9)**	61 (17.3)	62 (17.6)	229 (65.1)
	Female	159 (31.1)	23 (14.5)	33 (20.8)	103 (64.8)
**Risk factors** **	Diabetes	**121 (18.8)**	11 (9.1)	33 (27.3)	77 (63.6)
	Hypertension	**119 (18.5)**	12 (10.1)	31 (26.1)	76 (63.9)
	COVID-19	43 (6.7)	25 (58.1)	9 (20.9)	9 (20.9)
	COPD	41 (6.4)	11 (26.8)	7 (17.1)	23 (56.1)
	ESRD	19 (3.0)	1 (5.3)	2 (10.5)	16 (84.2)
	Cancer	2 (0.3)	0 (0.0)	2 (100)	0 (0.0)
	Immunocompromised	1 (0.2)	1 (100)	0 (0.0)	0 (0.0)
	No risk	**297 (46.2)**	42 (14.1)	47 (15.8)	208 (70.0)
**Sample Type**	Swab	**186 (36.4)**	26 (14.0)	30 (16.1)	130 (69.9)
	Blood	**182 (35.6)**	40 (22.0)	47(25.8)	95 (52.2)
	Urine	123 (24.1)	16 (13.0)	14 (11.3)	93 (75.6)
	Sputum	14 (2.7)	1 (7.1)	4 (28.6)	9 (64.3)
	CSF	6 (1.2)	1 (16.7)	0 (0.0)	5 (83.3)
**Status**	Infected	**291 (56.9)**	52 (17.9)	61 (21.0)	178 (61.2)
	Colonized	220 (43.1)	32 (15.0)	34 (15.5)	154 (70)
**Device association** **	Central Line	**346 (37.7)**	53 (15.3)	71 (20.5)	222 (64.2)
	Peripheral Line	96 (18.8)	8 (8.3)	9 (9.4)	79 (82.3)
	Peripherally inserted central catheter	8 (1.6)	8 (100)	0 (0.0)	0 (0.0)
	Foley Catheters	**439 (85.9)**	70 (16.0)	83 (19.0)	286 (65.2)
	Ventilation	**350 (68.5)**	52 (14.9)	64 (18.3)	234 (66.9)
	Tracheostomy	**275 (53.8)**	32 (11.6)	33 (12)	210 (76.4)
	Endotracheal Tubes	141 (27.6)	37 (26.2)	38 (27.0)	66 (46.8)
	Chest tube	23 (4.5)	8 (34.8)	7 (30.4)	8 (34.8)
**Antibiotics exposure**		435 (85.3)	64 (14.7)	71 (16.3)	300 (69.0)
**Region**	Central	**288 (56.4)**	--	--	--
	Eastern	57 (11.2)	--	--	--
	Western	**137 (26.8)**	--	--	--
	Southern	15 (2.9)	--	--	--
	Northern	14 (2.7)	--	--	--

*Total number of cases less than the sample size as there were 12 cases with missing data regarding age/

**Total number of cases was more than the sample size as some cases had multiple diagnoses, risk factors, or device associations.

The outcome of the cases evaluated during the outbreak is shown in **Table 2.** The general mortality rate for infected individuals (44.7%) was non-significantly higher than colonized patients (37.3%) at (*p value* = 0.093). The overall general mortality rate among the study cohort was 41.5%.

**Table 2:** Outcome of the studied cases of *Candida auris* during the outbreak.

Outcome	Infected	Colonized	Total	P value
	n = 291 (%)	n = 220 (%)	n = 511 (%)	
**Deceased**	130 (44.7)	82 (37.3)	212 (41.5)	0.093
**Alive**	161 (55.3)	138 (62.7)	299 (58.5)	

**Table 3** shows the characteristics of *Candida auris* infected and colonized patients. The prevalence of hypertension (p = 0.001), DM (p = 0.003), and peripheral line insertion (p = 0.004) was significantly higher among colonized patients. While the presence of COVID-19 (p = 0.001), and central line insertion (p = 0.001) was significantly higher among infected patients.

**Table 3:** Comparison between the clinical characteristics of *Candida auris* infected and colonized cases during the outbreak.

Parameter		Colonized	Infected	P-value
		N = 220 (%)	N = 291 (%)	
Gender Male (352)		160 (72.73)	192 (65.98)	0.103
Age ≥65 years (164)		70 (32.6)	94 (33.1)	0.899
Risk factors	Cancer (2)	2 (1.0)	0 (0.0)	NA
	COPD (41)	13 (5.9)	28 (9.6)	0.107
	COVID-19 (43)	5 (2.3)	**38 (13.1)**	0.001*
	Diabetes (121)	**66 (30.0)**	55 (18.9)	0.003*
	ESRD (19)	10 (4.5)	9 (3.1)	0.390
	Hypertension (119)	**67 (30.5)**	52 (17.9)	0.001*
	Immunocompromised (1)	1 (0.5)	0 (0.0)	NA
Device association	Central Line	130 (59.09)	**216 (74.23)**	0.001*
	Peripheral Line	**54 (24.55)**	42 (14.43)	0.004*
	Peripherally inserted central catheter	4 (1.82)	4 (1.37)	0.689
	Mechanical Ventilation	155 (70.45)	195 (67.01)	0.407
	Chest tube	8 (3.64)	15 (5.15)	0.412
	Tracheostomy	124 (56.36)	151 (51.89)	0.315
	Foley Catheters	193 (87.73)	246 (84.54)	0.305
	Endotracheal Tubes	55 (25.0)	86 (29.55)	0.254
Antibiotics exposure		183 (83.18)	252 (86.60)	0.283

*P value ≤0.05 is considered statistically significant. NA; Not applicable.

### Role of infection control measures in controlling the outbreaks

When a case of *Candida auris* was confirmed in a hospital, immediate notification to the infection control department, nurse in charge in the patient ward, nursing manager, and housekeeping manager was done.

The infection prevention and control team at each hospital began the following activities to contain the outbreak and prevent the spread of infection in the facility according to the GDPIC’s infection prevention and control recommendations for candida auris:

### Standard and contact precautions

- Patient placement in a single room with a private bathroom with Strict Contact precautions following. Patients with C. auris were placed with other patients with the same organism owing to the presence of multiple cases and a limited number of single rooms in some hospitals.
- Training for healthcare workers for proficiency and competency in the use of PPE and hand hygiene practice and strict monitoring to adherence to infection control measures.
- Dedicating the nursing staff to caring for patients with C. auris. If multiple C. auris patients are present in a facility, cohorting staff who care for these patients is done.
- Utilizing a dedicated isolation cart to keep all routine patient care supplies outside of the isolation room. Also, the dedication of patient care equipment occurs in case of the absence of disposable ones. All non-dedicated and non-disposable medical equipment used for confirmed or suspected cases should be cleaned and disinfected twice for extra precaution, using the recommended hospital-approved disinfectants and following the manufacturer’s instructions.
- Entry of visitors to the patient’s room was restricted.

### •Environmental Cleaning

The IPC team noticed that environmental decontamination in some of hospitals was done with the routinely used inadequate disinfectants e.g Quaternary Ammonia Compounds. To maintain clean rooms and high-touch surfaces, the proper dilution and use of disinfectants with a proven sporicidal effect for C. auris were emphasized.

- A solution of 1:50 household bleach for three times daily general cleaning and a solution of 1:10 for terminal cleaning was used with contact time of ten (10) minutes.
- The 1:50 household bleach chlorine disinfectant solution was used for cleaning and disinfection of areas outside of their rooms where they receive care (e.g., radiology, physical therapy).
- Housekeepers performing environmental cleaning should wear the recommended PPEs.
- Using designated cleaning equipment (e.g., mop, buckets, etc.) and disposable cleaning materials in the isolation room/unit.
- Environmental screening was performed by the IP&C department.
- Checklist and audit tools assist supervisory staff in monitoring and documenting cleaning and disinfection of environmental surfaces and medical equipment.

### •Monitoring and Management of Potentially Exposed Staff and patients

- HCWs who had a direct and prolonged interaction with the positive case 4 weeks before identification was screened using a composite swab for axilla and groin and nares.
- Once a member of staff is colonized with C. auris, he/she is off from work and referred to Surveillance or Staff Clinic for assessment and recommendation. Moreover, screening all patients in the unit was done if the patient was housed for more than 3 days undiagnosed.

### • Decolonization Protocol for 5 days

1. 4% Chlorhexidine gluconate CHG soap twice a day is used to reduce or inhibit skin colonization.
2. 0.2% Chlorhexidine mouthwash is used for patients on ventilators.
3. Oral Nystatin if oropharyngeal is colonized.

### • Screening strategy

For all patients hospitalized to the intensive care unit or with particular risk factors, screening was directed to all facilities that were experiencing outbreaks. Four weeks before finding the index case, the medical facility should review for an increased detection of candida auris, as this could indicate unrecognized transmission.

## Discussion

*C*. *auris* is a multidrug-resistant fungus capable of causing invasive infections worldwide. We present *C*. *auris* infections from Saudi Arabia MOH hospitals for three years (2020–2022). Table 1 shows that more than half of our study individuals (56.9%) were considered infected cases found through active screening upon request and (43.1%) were colonized. The inability to detect candida auris infection may be due to a lack of diagnostic kits, a longer time for the *C*. *auris* culture result, inaccuracy in identifying candida species, and some gaps in infection prevention practices, most notably ineffective cleaning of surfaces and equipment and adherence to infection control measures.

Our data suggest that *C*. *auris* could spread within the hospital setting. According to Table 1 and Fig 1, the majority of patients in the current study were admitted to the ICU (95.5%) and had a central venous catheter (37.7%), a urinary catheter (85.9%), a mechanical ventilator (68.5%), and a tracheostomy (53.8%), which all corroborate this hypothesis and are consistent with Caceres et al., 2019 and Amer et al., 2023 [20, 21]. Infections with *C*. *auris* appear to be hospital-acquired, occurring many weeks into a patient’s hospital stay, indicating an exogenous rather than endogenous source and a violation of infection control standards.

***Table 1*** shows that the most common comorbidities reported by our study individuals are diabetes (18.8%) and hypertension (18.5%), followed by COVID-19 (6.7%) and COPD (6.4%). Diabetes and hypertension are common in the Saudi population. Given the complexities of such chronic conditions that may need ICU admission, as well as the immunosuppressive effect of diabetes on the immune system, those candidates are mostly immunocompromised and sensitive to fungal infections such as *C*. *auris* [21].

At p = 0.001, Table 3 shows that (13.1%) of infected cases had confirmed COVID-19 infections versus (2.3%) of colonized cases. Some research has looked into the development of fungal infections, particularly multidrug-resistant types/species, in the COVID-19 era. Chowdhary et al., 2020 emphasized in their review of *C*. *auris* among critically ill COVID-19 cases in India that candidemia in those patients was usually caused by *C*. *auris* rather than *C*. *albicans* [22].

Previous studies identified considerable antibiotic exposure before *Candida auris* diagnosis as a significant risk factor for infection [23, 24]. The latter was consistent with our findings, as the majority of the cases evaluated (85.3%) had been exposed to antibiotics.

In the current investigation, the overall mortality rate was 41.5%, and it was greater among infected patients (44.7%) than colonized cases (37.3%), as shown in Table 2. The preceding conclusion was consistent with the findings of these studies: 50.7% in Kuwait [25], 50% in India [26], 46% in South Africa [27], and 43.4% in Saudi Arabia [21].

This expansion in *C*. *auris* infections has been rationalized by resource constraints like staff shortages and an insufficient supply of personal protective equipment during the pandemic, which impacts adherence to important infection control techniques. Inadequate hand hygiene, contamination of the surrounding environment, usage of shared medical equipment, and prolonged use of gowns and gloves are some of the infection control gaps that likely contributed to *C*. *auris* transmission [28, 29]. The previous was supported in our research by the fact that containment of the outbreak occurs immediately after strict adherence to IPC guidelines was followed by the worked staff.

The findings of our investigation highlight the importance of adhering to infection control recommendations, particularly aspects of careful central line care and maintenance, hand hygiene, proper disinfection of medical equipment, and use of standard and contact precautions, as well as raising awareness among healthcare workers about *C*. *auris* (habitat, resistance, detection, mode of transmission, and prevention, etc.…). Furthermore, it is vital to emphasize that *C*. *auris* must be identified using specialized laboratory methods. Traditional laboratory procedures may result in patient misidentification and incorrect care, making it difficult to limit the spread of *C*. *auris*.

Because of the investigation’s retrospective nature, the current study has some limitations. First, each patient had just a limited amount of clinical data. The most frequently missing data were prior antifungal medication exposure, the extent of antibiotic use, hospitalization period, and laboratory values. Second, we were unable to identify risk factors specific to *C*. *auris* because we lacked a reference group of patients with other Candida infections or healthy controls from the same institutions. Third, genetic testing for *C*. *auris* strains is not available at all hospitals.

In conclusion, candida auris is a substantial transmissible and nosocomial pathogen that causes a wide range of invasive infections and has a high mortality rate. Source control is the most effective therapeutic option for containment of the outbreaks. Molecular screening is becoming increasingly popular in diagnosis due to its rapid results. Commercial MALDI-TOF MS identification systems contain reliable spectra in their databases.

Based on previous findings we recommend strict adherence to the policy of the National Center for Disease Prevention and Control and GDIPC involving the updated *C*. *auris* guidelines and patient screening policy. One-month retrospective surveillance following diagnosis of the index case for high-risk patients to detect any missed cases. Building the capacity of MOH microbiology labs and providing diagnosed machines in all hospitals and reference labs is highly recommended. Training and education of HCWs regarding the *C*. *auris* prevention strategy and strict implementation of environmental cleaning in hospitals is mandatory. Furthermore, it is highly recommended to conduct prospective surveillance for a minimum of three months following the identification of the index patient or, in cases where transmission is detected through surveillance or screening, three months after the last identified case.

## Abbreviations

HA: Hospital-acquired
MOH: Ministry of Health
CDC: Center of Disease Control
GDPIC: General Directorate of Infection Prevention and Control/ MOH
C.: auris: Candida auris
COPD: Chronic obstructive pulmonary disease
IPC: Infection prevention and control
CSF: Cerebrospinal fluid
ICU: Intensive care unit
IFD: Invasive Fungal Disease
CLSI: Clinical Laboratories Standards institute
BMD: Broth Microdilution
